# Chemical Linkage to Injected Tissues Is a Distinctive Property of Oxidized Avidin

**DOI:** 10.1371/journal.pone.0021075

**Published:** 2011-06-20

**Authors:** Rita De Santis, Anna Maria Anastasi, Angela Pelliccia, Antonio Rosi, Claudio Albertoni, Antonio Verdoliva, Fiorella Petronzelli, Valeria D'Alessio, Serenella Serani, Carlo Antonio Nuzzolo

**Affiliations:** 1 Department of Immunology, Sigma-Tau SpA, Pomezia, Rome, Italy; 2 Department of Development, Tecnogen SpA, Piana di Monte Verna, Caserta, Italy; Anne Arundel Community College, United States of America

## Abstract

We recently reported that the oxidized avidin, named AvidinOX®, resides for weeks within injected tissues as a consequence of the formation of Schiff's bases between its aldehyde groups and tissue protein amino groups. We also showed, in a mouse pre-clinical model, the usefulness of AvidinOX for the delivery of radiolabeled biotin to inoperable tumors. Taking into account that AvidinOX is the first oxidized glycoprotein known to chemically link to injected tissues, we tested in the mouse a panel of additional oxidized glycoproteins, with the aim of investigating the phenomenon. We produced oxidized ovalbumin and mannosylated streptavidin which share with avidin glycosylation pattern and tetrameric structure, respectively and found that neither of them linked significantly to cells *in vitro* nor to injected tissues *in vivo*, despite the presence of functional aldehyde groups. The study, extended to additional oxidized glycoproteins, showed that the *in vivo* chemical conjugation is a distinctive property of the oxidized avidin. Relevance of the high cationic charge of avidin into the stable linkage of AvidinOX to tissues is demonstrated as the oxidized acetylated avidin lost the property. Plasmon resonance on matrix proteins and cellular impedance analyses showed *in vitro* that avidin exhibits a peculiar interaction with proteins and cells that allows the formation of highly stable Schiff's bases, after oxidation.

## Introduction

We recently reported that a ligand-protected oxidized avidin (OXavidin_HABA_, AvidinOX®) exhibits the property to reside within injected tissues for weeks thus constituting a stable artificial receptor for biotinylated therapeutics [1]; [2]. We also showed, in a mouse model, the use of this product for the targeting of radiolabeled biotin to inoperable neoplastic tissues, resulting in eradication of cancer lesions [3]. Tissue residence of AvidinOX was found to be dependent on the formation of Schiff's bases between avidin aldehyde groups, generated by sodium periodate oxidation of the sugar pyranosidic rings, and tissue protein amino groups. In fact, conversion of aldehyde groups to semicarbazones by reaction with semicarbazide resulted in reduced tissue residence [1]. Oxidation of glycoproteins with sodium periodate is the first step of a laboratory procedure used for decades to generate conjugates *in vitro* [4]; [5]. For example, to produce enzyme linked immunoglobulins, aldehyde groups derived from oxidation of the sugar pyranosidic rings of a given antidody, are condensed with the amino groups of an enzyme. The condensation reaction, resulting in the formation of Schiff's base, must be performed at neutral/basic pH because at acidic pH amino groups are in the inert protonated NH_3_^+^ status. However, while the formation of stable adducts between oxidized sugars and protein amino groups is a common event *in vivo*, leading to protein glycation [6], AvidinOX is the first example of adduct formation between an oxidized glycoprotein, which is formulated at acidic pH to prevent homologous reaction, and tissue proteins.

The aim of the present work was to investigate such *in vivo* chemical conjugation and we included in the study several oxidized glycoproteins. Surprisingly, we found that the stable linkage to the injected tissues is a distinctive property of AvidinOX being not exhibited by the other oxidized glycoproteins tested. Our analysis demonstrates that the peculiar electrostatic interaction of the highly cationic avidin with negatively charged biological substrates is an essential condition for the formation of Schiff's bases of the oxidized derivative *in vivo.*

## Results

### Tissue residence and cell binding of oxidized ovalbumin

**Figure 1:**
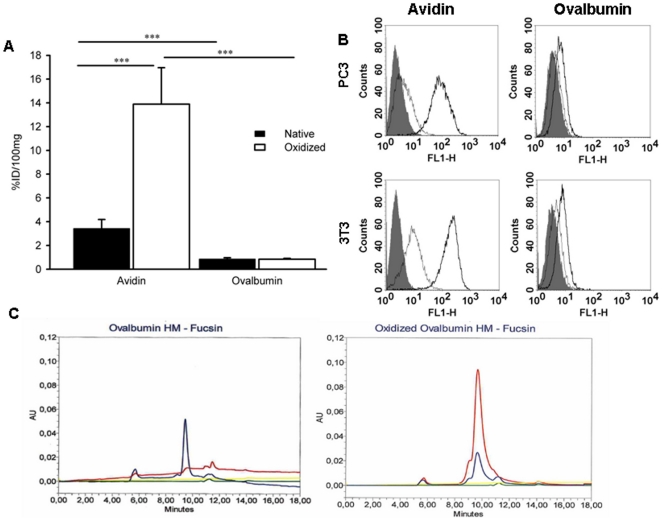
Tissue residence and *in vitro* cell binding of native and oxidized avidin and ovalbumin. **A,** Avidin and ovalbumin were ^125^I-labeled and then oxidized with sodium periodate. After purification, native and oxidized samples were diluted at 3.0 mg/ml in sodium acetate pH 5.5 and 15 µl were injected intramuscularly in one hind limb of mice. After 24 hours, the mice were sacrificed and treated limbs collected, weighed and counted in a gamma counter. Data are expressed as the % of injected dose/100 mg (% ID/100 mg) of tissue. Each point is the average of 5 mice. Bars represent standard deviation. Statistical analysis by Student's t test. ***p<0.001. **B**, Cytofluorimetry of the binding of native and oxidized avidin and ovalbumin to human prostate carcinoma PC3 and mouse fibroblast NIH-3T3 cells. Cells were incubated with 150 µM of either native or oxidized avidin or ovalbumin and binding revealed by specific primary antibodies as detailed in Materials and Methods. Gray peaks represent background (second antibody, only). Thin and bold lines, native and oxidized glycoproteins, respectively. **C**, Size exclusion chromatography on Bio-Sep-SEC-S 3000 (Phenomenex) of ovalbumin (left panel) or oxidized ovalbumin (right panel) after reaction with pararosaniline dye (Schiff's reagent, Sigma-Aldrich, USA). Baseline absorbance of dye at 280 and 550 nm (yellow and green lines, respectively) and ovalbumin or oxidized ovalbumin absorbance peaks at 280 nm (blue) and 550 nm (red). Red peak in the right panel indicates the formation of Schiff's bases between oxidized ovalbumin and the pararosaniline dye thus confirming the presence of functional aldehydes on oxidized but not native (left panel) ovalbumin.

1AvidinOX is the first glycoprotein described to chemically link to injected tissues. Therefore, to evaluate the extent of the phenomenon, we evaluated other oxidized glycoproteins. We started with the oxidation of the chicken ovalbumin [7] that, as chicken avidin, is extracted from the egg white and exhibits the same glycosylation pattern (high mannose and N-acetylglucosamine hybrid sugars in bi-antennary bisected and non-bisected structures). However, avidin is a glycoprotein with an isolectric point (pI) >10 and a 66 kDa tetrameric structure [8] while ovalbumin is a monomeric protein of 45 kDa with pI 4.7. Oxidation with 20 mM sodium periodate of ^125^Iodine labeled avidin and ovalbumin, produced 16.5 and 12.6 aldehyde groups/glycoprotein, respectively. Radiolabeled avidin and ovalbumin, in their native and oxidized forms, were intramuscularly injected in mice and surprisingly, it was found that, 24 hours after injection, the amount of oxidized avidin in the tissue was about 20 times that of oxidized ovalbumin (Fig. 1A). Notably, native avidin persisted longer in the limb muscle than native ovalbumin, thus suggesting an intrinsic property of the former to interact with tissues.

To further investigate this result we evaluated by cytofluorimetry the cell binding of oxidized avidin and ovalbumin. Human prostate carcinoma PC3 and mouse fibroblast NIH-3T3 cells were selected as model cell lines. Consistently with *in vivo* data, only native avidin but not native ovalbumin exhibited a non specific binding to cell membranes of both cell lines and this binding was significantly increased after oxidation while binding of oxidized ovalbumin was barely detectable (Fig. 1B).

Reactivity of the aldehydes of oxidized ovalbumin was confirmed by chromatography that showed the formation of adducts with the dye pararosaniline (Schiff's reagent) (Fig. 1C). Both *in vivo* and *in vitro* data indicated that oxidized avidin stably binds tissues and cells while oxidized ovalbumin does not despite functional aldehydes.

### Tissue residence and cell binding of oxidized glycosylated streptavidin

**Figure 2:**
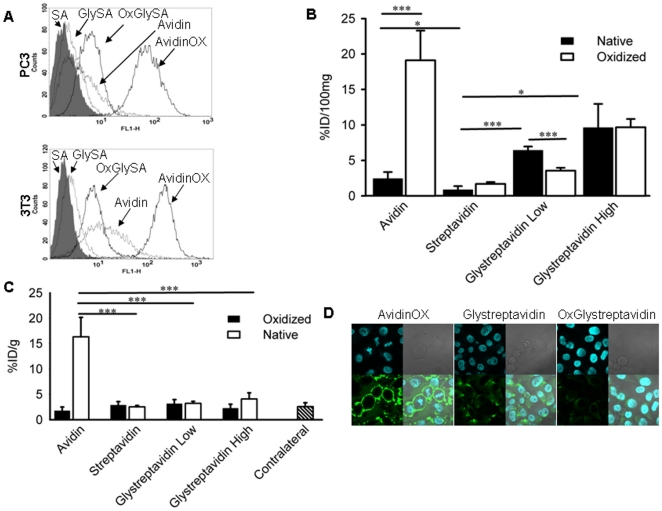
*In vitro* cell binding, *in vivo* tissue residence and biotin uptake of chemically glycosylated and oxidized streptavidin. **A**, Binding of streptavidin (SA), low mannose streptavidin (GlySA) and oxidized derivative (OxGlySA) to human prostate carcinoma PC3 and mouse fibroblast NIH-3T3 cells was compared to avidin and AvidinOX. Cells were incubated with 150 µM of either native or oxidized glycoproteins at +4°C and binding revealed by cytofluorimetry with specific primary antibodies as detailed in Materials and Methods. Gray peaks represent background (second antibody, only). Thin and bold lines, native and oxidized glycoproteins, respectively. **B**, Avidin, streptavidin, low and high mannose streptavidin (corresponding to 26 and 60 % of mannosylated amino groups, respectively) were ^125^I-labeled and oxidized with sodium periodate as detailed in Materials and Methods. All samples in their native and oxidized form were diluted to 3.0 mg/ml in sodium acetate pH 5.5 and 15 µl were injected intramuscularly in one hind limb of mice. After 24 hours, the mice were sacrificed and treated limbs collected, muscle weighed and counted in a gamma counter. Data are expressed as the % of injected dose/100 mg (% ID/100 mg) of tissue. Each point is the average of 5 mice. Bars represent standard deviation. Statistical analysis was performed by Student's t test. ***p<0.001; *p<0.05. **C**, **^111^**In-ST2210 was intravenously injected, 24 hours after the intramuscular administration of 15 µl of AvidinOX or indicated substances, at 3 mg/ml, in one hind limb of Balb/c mice. Mice were sacrificed after 2 hours and pre-treated and contra lateral limbs muscle (average from all treated groups) weighed and counted in a gamma counter. Data are expressed as the % of injected dose/g (% ID/g) of tissue. Each point is the average of 5 mice. Bars represent standard deviation. Statistical analysis by Student's t test. ***p<0.001. **D**, Confocal microscopy of PC3 cells incubated with the indicated products at 37°C as described in Materials and Methods. Mounting medium (Vectashield, Vector) containing DAPI (H-1200) was used to stain nuclei. Each panel shows DAPI stained nuclei (upper left), phase contrast image (upper right), staining with primary antibodies followed by second FITC-conjugated antibodies (lower left), merge of DAPI and avidin/streptavidin staining (lower right).

Trying to explain the previous unexpected result, we assessed whether the spatial position of aldehydes on the tetrameric structure of avidin might account for its interaction with tissue protein amino groups leading to the formation of Schiff's bases. Streptavidin [9] is an acidic (pI 5-6) non glycosylated protein with a tetrameric organization homologous to that of avidin as previously shown by superimposition of avidin and streptavidin crystallographic structures [10]. Taking into account the high mannose glycosylation pattern of avidin, streptavidin was chemically conjugated to mannose obtaining two derivatives with 26 % (low mannose) and 60 % (high mannose) mannosylated amino groups, respectively. Cytofluorimetry of PC3 and NIH-3T3 cells showed that the binding of low mannose streptavidin and oxidized low mannose streptavidin (8.5 aldehydes/glycoprotein) to PC3 and NIH-3T3 cells after 1 hour at 4°C was lower than native and oxidized avidin, respectively (Fig. 2A). Similar results were obtained with oxidized high mannose streptavidin exhibiting 11.1 aldehydes/glycoprotein (data not shown).

To evaluate the residence *in vivo*, both low and high mannose ^125^I-streptavidin were oxidized after saturation with HABA, as previously described for avidin [1]. When streptavidin and low and high mannose streptavidin were injected intramuscularly in one hind limb of mice, 0.85±0.54, 6.42±0.54 and 9.61±3.36 % ID/100 mg of tissue were found after 24 hours, respectively, compared to 2.41±0.95 % ID/100 mg of native avidin. The oxidized streptavidin and oxidized low and high mannose streptavidin were 1.69±0.25, 3.55±0.41 and 9.68±1.16 % ID/100 mg of tissue, respectively, compared to 19.11±4.19 of AvidinOX. Both low and high mannose streptavidin exhibited a higher tissue residence compared to streptavidin, but interestingly, their oxidized derivatives showed reduced (low mannose) or equal (high mannose) tissue residence compared to their native form (Fig. 2B). In parallel groups of mice the same products were injected intramuscularly and, after 24 hours, ^111^In-biotinDOTA (ST2210) was intravenously administered. The radioactivity uptake in the limb treated with streptavidin, glycosylated streptavidin or their oxidized derivatives was in any case lower than 4 % ID/g of tissue as compared to about 16 % ID/g of AvidinOX-treated limbs (Fig. 2C). In order to explain such lack of uptake of radiolabeled biotin by the tissue resident glycosylated streptavidin (oxidized or native) confocal microscopy was performed on PC3 and NIH-3T3 cells incubated at 37°C with the indicated proteins in culture medium as described in Materials and Methods. While AvidinOX was confirmed to strongly bind PC3 cell membranes, a clear intra-cytoplasmic localization of glycosylated and, to an apparent lower extent, oxidized glycosylated streptavidin was observed (Fig. 2D). Native avidin was also found inside the cells while native streptavidin was undetectable (Fig. S1). Same results were obtained with NIH-3T3 cells (data not shown). Confocal experiments showed that streptavidin, once mannosylated, is internalized by active endocytosis (dead or damaged cells show negative cytoplasm) indicating that the presence of sugars on such avidin like tetramer, enhances cell binding and tissue residence as compared to native streptavidin. However, confocal microscopy also clearly indicated that while oxidized avidin strongly binds to cell membranes, oxidized glycostreptavidin is actively translocated inside the cells thus explaining the lack of biotin uptake *in vivo*. Overall, the study indicates that the presence of sugars on a tetrameric structure resembling the avidin quaternary organization is necessary but not sufficient to explain the binding property of AvidinOX.

### Tissue residence and cell binding of oxidized glycoproteins and acetylated avidin

**Figure 3:**
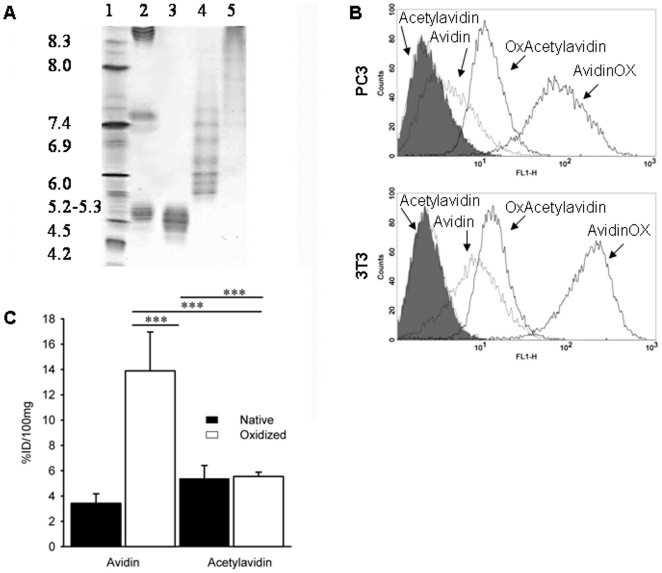
*In vitro* binding and *in vivo* residence of acetylated avidin. **A,** Isoelectric focusing of acetylated avidin. Lane 1, IEF standards; Lane 2, Herceptin, streptavidin and ovalbumin mixture; Lane 3, Acetylated avidin 1∶80; Lane 4, Acetylated avidin 1∶40; Lane 5, Acetylated avidin 1∶20. **B,** Cells were incubated with 150 µM avidin, AvidinOX, acetylated avidin pI 4.7 (Acetylavidin) or oxidized acetylated avidin (OxAcetylavidin) at +4°C and binding revealed by cytofluorimetry with an anti-avidin antibody as detailed in Materials and Methods. Gray peaks are background (second antibody, only). Thin and bold lines, native and oxidized glycoproteins, respectively. **C,** Avidin and acetylated avidin pI 4.7 (Acetylavidin), were ^125^I-labeled and then oxidized with sodium periodate as described in Materials and Methods. All samples were diluted at 3.0 mg/ml in sodium acetate pH 5.5 and 15 µl were injected intramuscularly in one hind limb of mice. After 24 hours, the mice were sacrificed and treated limbs collected, weighed and counted in a gamma counter. Data are expressed as the % of injected dose/100 mg (% ID/100 mg) of tissue. Each point is the average of 5 mice. Bars represent standard deviation. Statistical analysis by Student's t test. ***p<0.001.

To get a measure of the peculiarity of AvidinOX we extended the evaluation of tissue residence and cell binding to three additional oxidized glycoproteins namely PTX3 (multimeric, 10% of complex sugars, pI 4.7) [11], Herceptin® (chimeric immunoglobulin, 4% of complex sugars, pI 9.2) [12], carcinoembryonic antigen, CEA (monomeric, 50–60% of complex sugars, pI 3–4) [13] and found that none of them resided within injected tissues nor bound to cells *in vitro* like AvidinOX despite high number of aldehyde groups/glycoprotein (data not shown). We hypothesized that such peculiar behaviour of oxidized avidin might correlate with its uncommon high cationic charge. Therefore, to lower the pI, avidin was acetylated by reaction with acetic acid N-hydroxysuccinimide ester at 1∶20, 1∶40 and 1∶80 molar ratios. Isolectrofocusing analysis confirmed that 1∶80 reaction reduced the avidin pI to about 4.7 (Fig. 3A).

This acidic avidin was therefore oxidized as previously described generating 15.1 aldehyde groups/glycoprotein and tested for cell binding by cytofluorimetry. As shown in the Fig. 3B the acetylated avidin lost the cell binding property of native avidin while its oxidized derivative exhibited a lower binding compared to oxidized avidin. *In vivo* data showed for the acetylated and oxidized acetylated avidin a tissue residence, 24 hour after intramuscular injection, slightly higher than that of native avidin but about 1/3 compared to oxidized avidin (Fig. 3C). These data lead to the conclusion that the cationic charge is relevant to the AvidinOX capacity to chemically link to cells and tissues.

### Protein and cell interaction analyses

**Figure 4:**
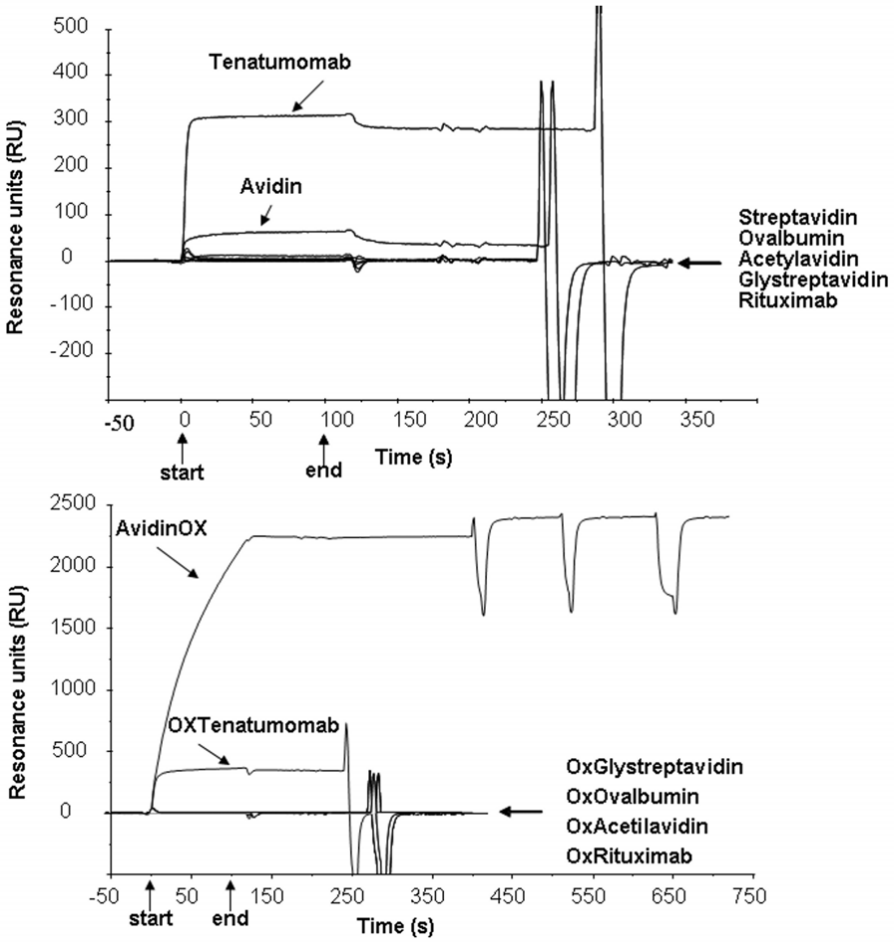
Interaction of AvidinOX and other native/oxidized glycoproteins with matrix proteins by Surface Plasmon Resonance. Samples were injected in HBS at 1 µM and allowed to interact with immobilized mixture of human tenascin and fibronectin matrix proteins (immobilization signal 4100RU) for 1 minute, then allowed to dissociate for 1 minute. Sensorgrams were obtained subtracting background signals of the uncoated reference cell. Upper panel: Native proteins. Lower panel: Oxidized glycoproteins. Arrows indicate start and end of injection. Data are representative of two experiments.

To further investigate the avidin interaction with protein and cellular substrates, we performed protein interaction analysis by plasmon resonance and measured cellular impedance variations. Biacore chip was coated with a mixture of tenascin and fibronectin matrix proteins. A signal of about 300 Resonance Units (RU) was observed with Tenatumomab (anti-tenascin monoclonal antibody) [14], as expected. Avidin exhibited a non specific binding of about 30 RU while other proteins including the Rituximab (anti-CD20 monoclonal antibody) [15], streptavidin, glycosylated streptavidin, ovalbumin and acetylated avidin did not give a detectable resonance signal (Fig. 4, upper panel).

Concerning the oxidized derivatives, streptavidin (<2 aldehyde groups/protein), ovalbumin (12.6 aldehyde groups/glycoprotein), Acetylavidin (15.1 aldehyde groups/glycoprotein), GlyStreptavidin (8.5 aldehyde groups/glycoprotein), Rituximab (29.9 aldehyde groups/glycoprotein) and Tenatumomab (11.5 aldehyde groups/glycoprotein), injected at neutral pH, which potentially allows the formation of Schiff's bases, did not show binding as the related native forms. Surprisingly, more than 2000 RU signal was observed with AvidinOX (16.5 aldehyde groups/glycoprotein) indicating extensive linkage to the matrix proteins (Fig. 4, lower panel). Such linkage was proved to be irreversible: in fact, AvidinOX did not dissociate even after repeated attempts of chip regeneration thus leading to the conclusion that formation of highly stable Schiff's bases between AvidinOX and matrix proteins on the chip had occurred. The analysis was repeated on a new coated chip at acidic pH at which matrix proteins are in the inert protonated status. In this case avidin and AvidinOX gave a measurable binding that was easily reversed by chip regeneration. The system was then brought to neutral pH where the reversible binding of avidin and Tenatumomab was observed, confirming integrity of matrix proteins at low pH. As previously observed, injection of AvidinOX at neutral pH, resulted in its irreversible linkage to the coated chip thus confirming the occurrence of Schiff's bases (data not shown).

**Figure 5:**
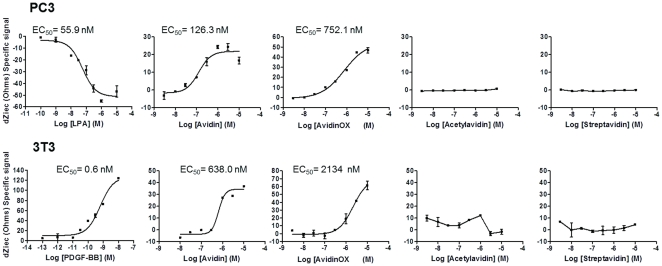
Interaction of AvidinOX and others glycoproteins with cells by Cellular Impedance analyses. Impedance assays on PC3 and NIH-3T3 cell lines by Cell Key® technology. All samples were tested up to 10 µM concentration. Impedance variation was recorded for 11 minutes after the addition of the samples. Background values were obtained from cells incubated with 0.1% BSA HBSS buffer and were subtracted from total signal.

To evaluate the effect of interaction of avidin and AvidinOX with cellular substrates we measured cellular impedance, a parameter known to be strictly related to responses of the leaving cells to different physico/chemical or mechanical stimuli [16]. Impedance analysis on PC3 cells indicated that the specific lysophosphatidic acid (LPA) receptor agonist [17] induced, in the 11 minutes after addition, a reduction of the signal with an EC50 of 55.9 nM. Interestingly, avidin and AvidinOX induced an increase of impedance with EC50 of 126.3 and 752.1 nM, respectively. Acetylavidin and streptavidin had no significant effect up to 10 µM (Fig. 5, upper panels).

These PC3 impedance results were confirmed on NIH-3T3 cells where the specific receptor ligand platelet-derived growth factor BB (PDGF-BB) [18] induced an impedance variation (increase of the signal) with an EC50 of 0.62 nM while avidin and AvidinOX induced also an increase of the signal with EC50 of 638 and 2134 nM, respectively. As for PC3, Acetylavidin and streptavidin had no effect up to 10 µM (Fig. 5, lower panels). No significant impedance variation on both cell lines was recorded with the highly cationic lysozyme (pI>10) [19] (Fig. S2) suggesting that the avidin cationic charge is necessary but not sufficient to explain induction of impedance variation.

## Discussion

Hen egg white avidin has high cationic charge (pI>10) and this feature has been previously related to its non specific binding to negatively charged proteins and cellular substrates and to tumor accumulation [20] and the present *in vitro* and *in vivo* data are in agreement with this observation. Nevertheless, we previously showed that the tissue half life of avidin is less than two hours [2].

Here we show that acetylated avidin, exhibiting acidic charge and not displaying basal binding to protein and cellular substrates, binds much less than AvidinOX to cells and tissues once oxidized. Present plasmon resonance data confirm the peculiar interaction of avidin with proteins, resulting in an irreversible linkage of AvidinOX but also indicate that binding is not a sufficient condition to allow the formation of a stable linkage as demonstrated by the reversibility of the binding of the oxidized Tenatumomab. Finally, cellular impedance analyses showed that avidin induces a clearly measurable change of electric potential most likely a consequence of its electrostatic interaction with cell membranes.

We might expect that other poultry avidins [21] as well as the chicken avidin-related protein 4/5 [22] or frog Xenavidin [23], all known to exhibit high cationic charge and structure similarity with avidin, might behave like AvidinOX, after oxidation. On the other end, besides streptavidin here investigated, other acidic avidins like bradavidins [24]; [25] or rhizavidin [26] might be investigated to further assess the role of protein charge and structure on the behaviour of oxidized derivatives.

On the overall, present data point to AvidinOX as a peculiar product whose *in vivo* property to link and reside within injected tissues for weeks is clearly related to its cationic charge that, by electrostatic interaction with negatively charged cellular membranes and extracellular proteins, promotes the formation of highly stable Schiff's bases. The use of this product for the targeted delivery of radiolabeled biotin will be clinically evaluated in the next future supported by the good safety and tolerability data obtained in rodents and non human primate studies [27].

## Materials and Methods

### Ethics Statement

The care and husbandry of the animals employed in the present studies were in accordance with the European Directive 86/609 and Italian legislation. The animal studies described in the present work were approved by the Italian Ministry of Health D.M. 137/2010-B.

### Periodate oxidation of avidin and other glycoproteins

Avidin (Tecnogen) was oxidized with 20mM sodium periodate (NaIO_4_) (Sigma- Aldrich), in 100 mM acetate at pH 5.5 for 1 hour at room temperature, in the presence of a molar excess of 4-hydroxyazobenzene-2′-carboxylic acid (HABA) (Sigma-Aldrich) and purified by chromatography on a Sephadex G-25 fine desalting column (GE Healthcare) as previously reported [3]. Ovalbumin (Sigma-Aldrich), pentraxin 3 (PTX3) (Tecnogen), CEA (US biological) Tenatumomab (Sigma-Tau), Rituximab (Roche Pharma) and Herceptin® (Roche) were oxidized (without HABA) with periodate and purified by size exclusion chromatography as described above for avidin. Glycosylated streptavidin was obtained by reacting streptavidin with α-D-mannopyranosylphenylisothiocyanate (Sigma-Aldrich) at 1∶25 molar ratio at +4°C for 5 or 24 hours and purified by size exclusion chromatography. The degree of glycosylation was evaluated by estimating the residual amino groups by reaction with 2,4,6-trinitrobenzene 1-sulfonic acid (TNBS) according to standard method [28]. Avidin acetylation was performed by reaction with acetic acid N-hydroxysuccinimide ester (Sigma-Aldrich) according to a described method [29] and the extent of amino group acetylation was determined by titration with TNBS as before. Oxidation of glycosylated streptavidin (Glystreptavidin) and acetylated avidin (Acetylavidin) was performed in the presence of HABA as previously described for avidin. The number of aldehyde groups (CHO) generated on the carbohydrate moieties by oxidation, was evaluated according to a published method [30] using Purpald® reagent (Sigma- Aldrich) and propionaldehyde diethyl acetal (Fluka) as a standard. Isoelectric point of glycoproteins was evaluated by isoelectrofocusing on 3-10 IEF gel (Invitrogen). Biotin binding capacity of strept/avidin and oxidized strept/avidin derivatives was evaluated by HABA assay according to standard method [31].

### Cytofluorimetry

Reactivity of primary antibodies towards native and oxidized glycoproteins was preliminary confirmed by ELISA. Cytofluorimetry was performed on mouse fibroblast NIH-3T3 and human prostate carcinoma PC3 cell lines (ATCC) after 1 hour incubation at 4°C with native or oxidized glycoproteins (150 µM/5×10^5^ cells in 100 µL of PBS). After washings, binding was revealed by incubating cells for 1 hour at 4°C with the following specific primary antibodies: mouse anti-avidin (Sigma-Aldrich), mouse anti-ovalbumin (Sigma-Aldrich) followed by FITC-conjugated goat anti-mouse Ig (Sigma-Aldrich); rabbit anti-streptavidin (Sigma-Aldrich) followed by FITC-conjugated goat anti-rabbit Ig (BD Pharmingen); rat anti-PTX3 monoclonal antibody (Alexis) followed by FITC-conjugated goat anti-rat Ig (BD Pharmingen); Herceptin® was revealed by FITC-conjugated mouse anti-human Ig (BD Pharmingen). Analysis was performed on a FACScalibur instrument and data elaborated by CellQuest software (Becton Dickinson).

### Confocal microscopy

Mouse fibroblast NIH-3T3 and human prostate carcinoma PC3 cell lines (1.0×10^5^) were plated on a coverglass and cultivated for 24 hours. Coverglasses were washed with serum free medium and incubated with 150 µM AvidinOX or oxidized glycosylated streptavidin or their related controls (native avidin, native streptavidin, glycosylated streptavidin) for 2 hours at 37°C in serum free medium. After washings with PBS, cells were fixed with 4% paraformaldehyde for 15 minutes at room temperature, washed with PBS, blocked with PBS, 1% BSA and incubated 1 hour at room temperature with primary anti-avidin or anti-streptavidin antibodies. After washings with PBS, coverglasses were incubated with FITC-conjugated second antibodies for 1 hour at room temperature as previously described for cytofluorimetry. After washings, coverglasses were observed at confocal microscopy Zeiss LSM 700.

### *In vivo* studies

Radiolabeling of all proteins was performed with ^125^Iodine by IODO-GEN™ (Pierce) before periodate oxidation. Radiolabeled compounds, formulated in 0.1 M sodium acetate pH 5.5, were injected in one hind limb muscle at the dose of 50 µg in 15 µl (about 50 kBq/mouse). At the indicated time points mice were sacrificed and the injected muscle weighed and radioactivity quantified by gamma counter. Each sample was referred to the injected dose that was re-counted at each time point to take into account radioactive decay. For the intra-tissue administration, data were expressed as the % of injected dose/100 mg of tissue (% ID/100 mg) to avoid values >100% being the treated muscle <1 g. The biotin-DOTA ST2210 (Sigma-Tau) was labelled with ^111^Indium according to the previously published method [32]. For uptake experiments, groups of mice were pre-treated with oxidized strept/avidin or strept/avidin in one hind limb and then injected intravenously, at the indicated time points, with 1 µg of ^111^In-ST2210 (about 300 kBq/mouse) in 200 µl of vehicle. Mice were sacrificed 2 hours after ^111^In-ST2210 injection and pre-treated limbs weighed and radioactivity quantified by gamma counter. Data were expressed as % ID/g of tissue.

### Plasmon resonance analysis

The interaction of glycoproteins and their related oxidized derivatives with matrix proteins was analyzed by BIAcore X (GE Healthcare). The CM4 sensor chip was prepared by immobilizing a mixture of human tenascin and human fibronectin (Tecnogen) through primary amino groups using amine coupling kit according to manufacturer's instruction (BIAcore AB). Immobilization gave a ΔRU = 4100. Each binding assay was performed in HBS (10 mM HEPES, 150 mM NaCl, 3 mM EDTA, 0.05% BIAcore surfactant P20 at pH 7.4) at the constant flow rate of 5 µl/minute, 25°C. Glycoproteins in their native and oxidized forms were injected at 1 µM concentration in HBS for 1 minute and then allowed to dissociate for 1 minute followed by regeneration of the sensor chip by a pulse of 0.1M NaOH. Sensorgrams were obtained by subtracting background signals from the reference uncoated cell and by elaboration with the BIAevaluation 4.1.1 software.

### Cell Key® analysis

Impedance assay was performed on PC3 and NIH-3T3 cells by using the Cell Key® technology (Cerep). Briefly, 3×10^4^ PC3 or 4×10^4^ NIH-3T3 cells were plated in 96 well microtiter plates and grown for 24 hours. Culture medium was then replaced by 0.1% BSA HBSS buffer added with the specific binders LPA (Sigma-Aldrich) or PDGF/BB (Sigma- Aldrich) for PC3 and NIH-3T3 cells, respectively or with serial dilution of avidin, AvidinOX, Acetylavidin (pI 4.7), streptavidin (BioSPA) or lysozyme (Sigma-Aldrich).

### Statistics

The results are expressed as mean ± S.D. Differences between groups were assessed using unpaired two-tailed Student's *t*-test or two-way analysis of variance (ANOVA) (factors of variance were dose and days of treatment). When normality test failed, it was used Kruskal-Wallis one-way analysis of variance on ranks (ANOVA on ranks). When the ANOVA manifested a statistical difference, all pairwise multiple comparison procedures (Dunnett's or Student- Newman-Keuls test) were applied. The p<0.05 value was considered to be significant in all experiments. Analysis of data and plotting of the figures were done with the aid of softwares (SigmaStat and SigmaPlot, Version 5.0, San Raphael, CA, USA; GraphPad PRISM®, Version 2.0, San Diego, CA, USA; Microsoft Excel 2003, Redmond, WA, USA).

## Supporting Information

Figure S1 **onfocal microscopy of PC3 cells incubated with avidin or streptavidin.** PC3 cells were incubated at 37°C with 150 µM of either avidin or streptavidin in culture medium as described in Materials and Methods. After processing, mounting medium (Vectashield, Vector) containing DAPI (H-1200) was used to stain nuclei. The four panels show: DAPI stained nuclei (upper left), phase contrast image (upper right), staining with primary antibodies followed by second FITC-conjugated antibodies (lower left), merge of DAPI and avidin/streptavidin staining (lower right).
(TIF)

Figure S2 **Impedance assays on PC3 and 3T3 cell lines incubated with lysozyme.** Lysozyme was tested up to 10 µM concentration. Impedance variation (Cell Key® technology) was recorded for 11 minutes after the addiction of the sample. Background values were obtained from cells incubated with 0.1% BSA HBSS buffer and were subtracted from total signal.
(DOC)
