# Supplementary figures and images for: Chemical Linkage to Injected Tissues Is a Distinctive Property of Oxidized Avidin
Source: PLoS One. 2011 Jun 20;6(6):e21075. doi: 10.1371/journal.pone.0021075 (PMC3118792; doi:10.1371/journal.pone.0021075)

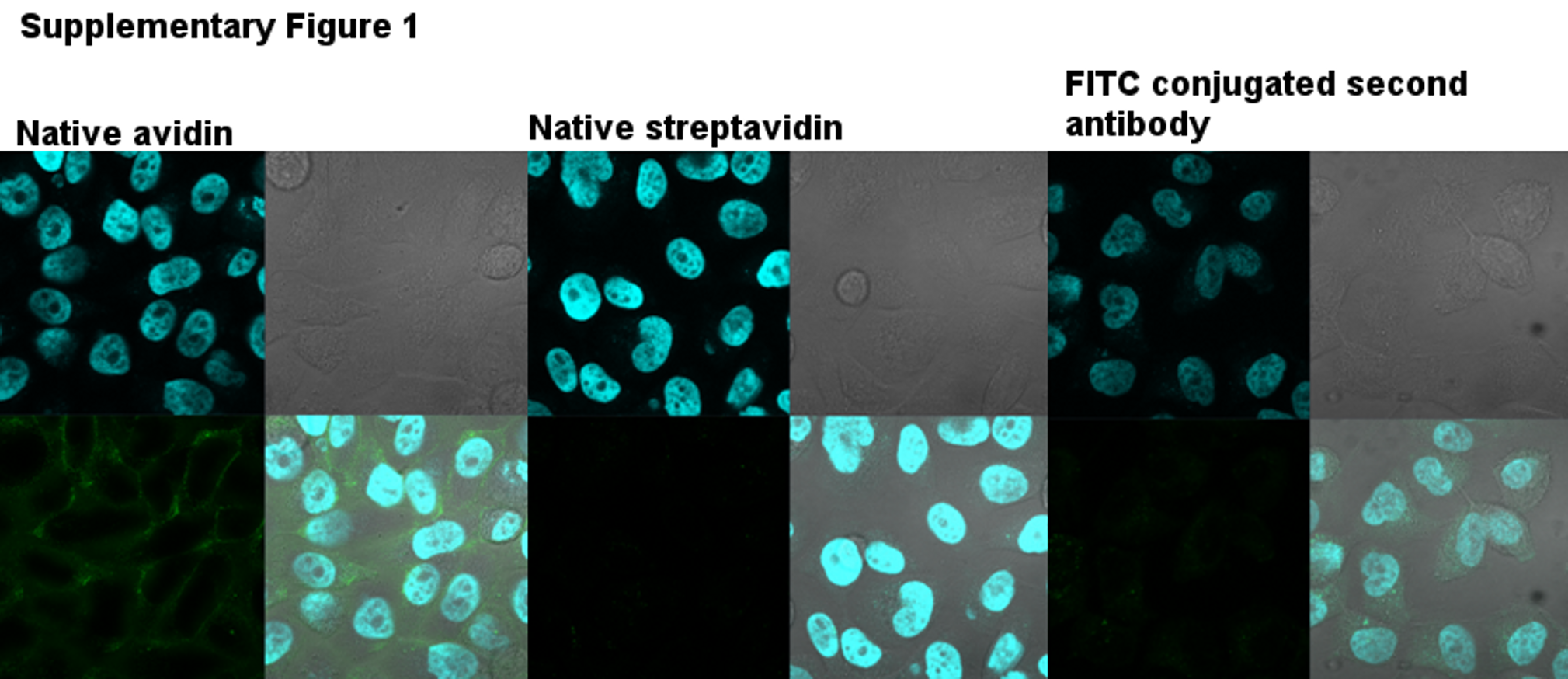

Supplement: Figure S1 — onfocal microscopy of PC3 cells incubated with avidin or streptavidin. PC3 cells were incubated at 37°C with 150 µM of either avidin or streptavidin in culture medium as described in Materials and Methods. After processing, mounting medium (Vectashield, Vector) containing DAPI (H-1200) was used to stain nuclei. The four panels show: DAPI stained nuclei (upper left), phase contrast image (upper right), staining with primary antibodies followed by second FITC-conjugated antibodies (lower left), merge of DAPI and avidin/streptavidin staining (lower right). (TIF) [file pone.0021075.s001.tif]
