# Supplementary material for: Chemical Linkage to Injected Tissues Is a Distinctive Property of Oxidized Avidin
Source: PLoS One. 2011 Jun 20;6(6):e21075. doi: 10.1371/journal.pone.0021075 (PMC3118792; doi:10.1371/journal.pone.0021075)
Supplement: Figure S2 — Impedance assays on PC3 and 3T3 cell lines incubated with lysozyme. Lysozyme was tested up to 10 µM concentration. Impedance variation (Cell Key® technology) was recorded for 11 minutes after the addiction of the sample. Background values were obtained from cells incubated with 0.1% BSA HBSS buffer and were subtracted from total signal. (DOC) [file pone.0021075.s002.doc]

**Supplementary Figure 2**

**PC3 cells**

**3T3 cells**
